# Prevalence of sexual dysfunction in men with multiple sclerosis: a systematic review and meta-analysis

**DOI:** 10.1186/s13643-020-01560-x

**Published:** 2021-01-06

**Authors:** Maryam Dastoorpoor, Maryam Zamanian, Rahmatollah Moradzadeh, Seyed Massood Nabavi, Raana Kousari

**Affiliations:** 1 Department of Biostatistics and Epidemiology, Social Determinants of Health Research Center, Ahvaz Jundishapur University of Medical Sciences, Ahvaz, Iran; 2 Department of Epidemiology, School of Health, Arak University of Medical Sciences, Arak, Iran; 3 Department of Brain and Cognitive Sciences, Royan Institute for Stem Cell Biology and Technology, ACCR, Royan, Iran; 4 Dezful University of Medical Sciences, Dezful, Iran

**Keywords:** Prevalence, Multiple sclerosis, Sexual dysfunction, Men, Systematic review

## Abstract

**Background:**

Symptoms in multiple sclerosis (MS) can lead to different types and ranges of sexual dysfunction in MS patients. Studies in different parts of the world have reported a high range of sexual dysfunction in men with MS. This study aimed to estimate pooled prevalence of sexual dysfunction in men with MS.

**Methods:**

The authors searched Web of Science, PubMed, Scopus, Embase, Magiran, SID, and Iran Medical Papers Database using the keywords “multiple sclerosis”, “sexual dysfunctions”, “men”, “prevalence”, and their synonyms systematically. Meta-analysis was performed using the random effects model with inverse variance-weighted method to estimate the overall prevalence of sexual dysfunction in men with MS. The protocol for this meta-analysis is available in PROSPERO (ID CRD42020199005).

**Results:**

A total of 351 documents were identified, and 20 articles published from 1996 to 2019 were analyzed. The articles used sample sizes from 9 to 101 individuals. However, two studies conducted online used 388 and 1568 samples. Prevalence of sexual dysfunction in all studies was reported from 31 to 92%, and the pooled prevalence of sexual dysfunction in men with MS in all studies was 62.9% with a 95% confidence interval 53 to 72.7% (heterogeneity: *I*^2^ = 96.3%, *Q*-statistic = 12.48, *P* value < 0.001). According to the results of Egger’s test, there was publication bias in the current study (*β* = 4.55, Se = 1.38, *P* value = 0.004).

**Conclusion:**

Sexual dysfunction is highly prevalent in men with MS. Diagnosing sexual dysfunction in MS patients in clinics by specialists have to be considered a necessity.

## Background

Multiple sclerosis (MS) is an autoimmune disorder mainly affecting young adults and is characterized by destruction of myelin in the central nervous system. Pathologic findings include multiple sharply demarcated areas of demyelination throughout the white matter of the central nervous system. Clinical manifestations include visual loss, extra-ocular movement disorders, paresthesias, loss of sensation, weakness, dysarthria, spasticity, ataxia, and bladder dysfunction. The usual pattern is one of recurrent attacks followed by partial recovery (multiple sclerosis, relapsing-relapsing). However, acute fulminating and chronic progressive forms (multiple sclerosis, chronic progressive) also occur [1].

Psychological sexual dysfunction is defined as disturbances in sexual desire and the psychophysiological changes that characterize the sexual response cycle and cause marked distress and interpersonal difficulty. Physiological sexual dysfunction is defined as physiological disturbances in normal sexual performance in both males and females [2, 3].

Sexual dysfunction can be divided into primary sexual dysfunction, in which an individual has never experienced sexual satisfaction, and secondary sexual dysfunction, in which former enjoyment of sexual activity is no longer present.

Developmental disorders of the neurological, endocrine, or urogenital system can be the cause of primary sexual dysfunction, and it may also have an underlying psychiatric cause. It does not seem that a developmental anomaly of the nervous, urogenital, or endocrine system be the cause of secondary sexual dysfunction. An acquired disorder, such as the cardiovascular disease, diabetes mellitus, obesity, depression, or anxiety is likely to be the cause [4]. Sexual dysfunction includes dyspareunia, erectile dysfunction, premature ejaculation, sexual and gender disorders, and vaginismus. Because of the significant relationship between sexual dysfunction and both physical and psychological disorders, MS symptoms can lead to different types and ranges of sexual dysfunction in MS patients [5].

Studies of different parts of the world have reported the rate of sexual dysfunction in MS patients such as 30%, 71%, and 73.1% [6]. Moreover, the rate of sexual dysfunction in men with MS is reported as 45.2%, 71%, 74%, 89%, and even 92.1% in some studies [6].

Sexual dysfunction is an essential component of quality of life, and sexual problems such as erectile dysfunction and ejaculation disorders have been shown to be significantly associated with decrease of life satisfaction, mood disorders, and quality of relationship [7]. Determination of sexual dysfunction prevalence in patients with MS can provide more clarification of these problems among patients. As the authors did not find any article that describes the prevalence of sexual dysfunction in adult men with MS in recent years, this study aimed to estimate pooled prevalence of all types of sexual dysfunction in adult men with MS.

## Materials and methods

### Protocol and registration

The study protocol was approved by the Ethics Committee of the Ahvaz Jundishapur University of Medical Sciences (IR.AJUMS.REC.1399.466), and it has been reported in the International Prospective Register of Systematic Reviews (PROSPERO) database (ID CRD42020199005).

### Identification and selection of studies

The literature was searched for identifying the available research studies that assessed sexual dysfunction in men with multiple sclerosis. We used these criteria to select eligible studies: (1) prevalence of sexual dysfunction was reported and (2) sexual dysfunction was reported separately for men. Studies that reported the indicators without gender segregation and studies that have reported indicators only in women were excluded, because they were not applicable to the scope of the current study. The studies of adult men (over 18 years old) were included. Studies with any form of sexual dysfunction were included (e.g., ED, impotence, retrograde ejaculation, delayed ejaculation). The cross-sectional studies were included. Moreover, original researches in the form of community-based studies, cross-sectional studies, observational cohorts, and case-control studies considering the prevalence of sexual dysfunction in MS patients were considered acceptable for inclusion.

### Search strategy

A systematic search of international literature databases and the Iranian literature databases was performed in December 2019 without time limitation with a research librarian. The international databases searched were Web of Science, PubMed, Scopus, and Embase, and the Iranian literature databases searched were Magiran, SID, and Iran Medical Papers Database. The relevant Medical Subject Heading (MeSH) terms and keywords “multiple sclerosis”, “Sexual dysfunctions”, “Men”, “Prevalence”, and their synonyms were used in various combinations using Boolean operators like “OR” or “AND”. The search was limited to papers published in English and Persian and humanities’ subjects because of time and resource limitation. To access more relevant papers, the researchers searched the reference lists of relevant literature manually. References were managed using EndNote X8.1.

### Data extraction

All titles, abstracts, and full-text articles were screened by two reviewers independently, MZ and RM. In case of disagreement, it was resolved by discussion, and in case of disagreement, a third independent party (MD) decided as an arbiter.

The items extracted by one reviewer were also checked for accuracy by another reviewer. The items extracted from each article included year of publication, country/city, sample size, response rate, age range and mean age, duration of MS, design of the study, the outcome of study, scale/index, the method of outcome assessment, and prevalence (*N* of event).

### Measure of research quality

The quality assessment was screened by two reviewers independently. In case of disagreement, it was resolved by discussion, and in case of further disagreement, a third independent party decided as an arbiter. We assessed the quality of studies through the “Quality Assessment Checklist for Prevalence Studies.” The total score of 0–3, 4–6, and 7–9 indicate the low, moderate, and high risk of bias, respectively. Based on the checklist, the studies with a high risk of bias were excluded [8].

### Statistical analyses

Meta-analysis was performed using the random effects model with the inverse variance-weighted method to estimate the overall prevalence of sexual dysfunction in men with MS. *I*^2^ was used to assess the studies’ heterogeneity. Therefore, meta-regression was used to determine potential sources of heterogeneity, and items used in the meta-regression included mean age, sample size, mean duration of MS, and the Expanded Disability Status Scale (EDSS) score. Egger’s test was used to evaluate the publication bias. Stata software 12 was used for data analysis.

## Results

A total of 351 documents were identified through all databases and search methods. After screening the titles, 139 documents were identified as duplicates, and 212 documents were screened through reading abstracts. After screening the abstracts, 89 records were assessed for eligibility through a comparison of their full text to inclusion and exclusion criteria of the study or the relevancy. Accordingly, 69 documents were excluded and a total of 20 articles were included in the systematic review (Fig. 2).

**Fig. 1:**
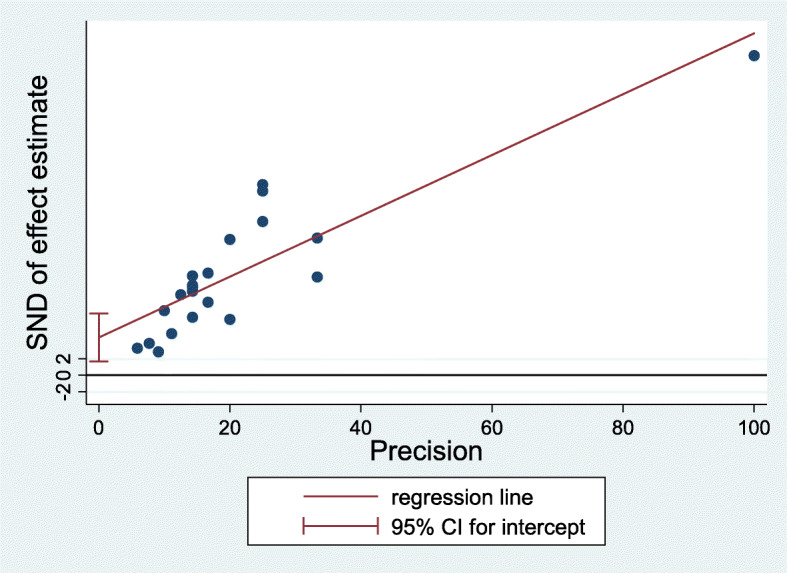
Egger’s publication bias plot

**Fig. 2:**
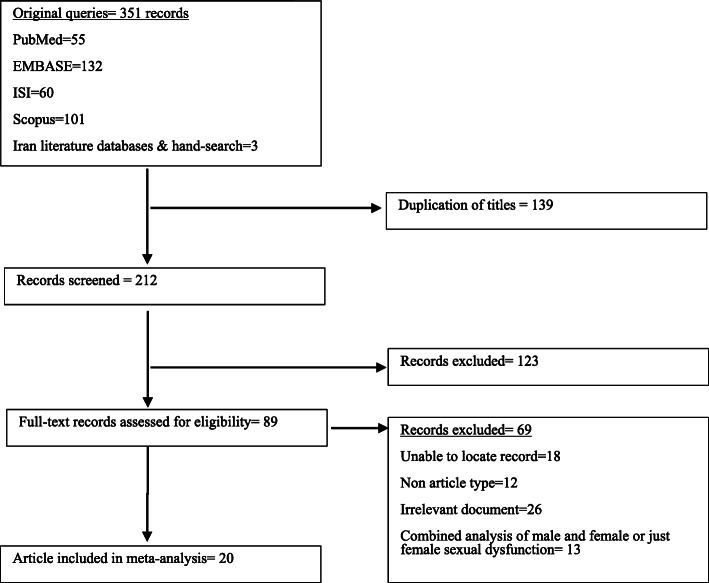
Database search and record screening

In this study, the results of the meta-analysis showed heterogeneity between studies (*I*^2^ = 96.3%, *Q*-statistic = 12.48, *P* value < 0.001). Meta-regression that was used to determine the potential sources of heterogeneity did not show statistically significant effect of the included items: mean age, sample size, mean duration of MS, and EDSS score (Table 1). According to the results of Egger’s test, there was publication bias in the current study (*β* = 4.55, Se = 1.38, *P* value = 0.004) (Fig. 1). The results of Pearson’s correlation coefficient showed that there is no significant relationship between the prevalence of sexual dysfunction and EDSS (*r* = − 0.04, *P* value = 0.904), patients’ age (*r* = 0.08, *P* value = 0.727), and duration of disease (*r* = 0.02, *P* value = 0.932) which were, respectively, reported in 14, 20, and 20 studies (Fig. 2).

**Table 1:** Meta-regression result to determine potential sources of heterogeneity

Variable	***β***	SE	***T*** value	***P*** value
EDSS	− 0.062	0.084	− 0.74	0.237
Disease duration (year)	0.041	0.032	1.27	0.237
Age of the patients	− 0.020	0.0198	-1.04	0.326
Sample Size	− 0.001	0.001	− 1.44	0.183

The main findings of 20 articles included in the current study are reported in Table 2. These 20 articles were published from 1996 to 2019 and assessed sexual dysfunctions in adult men with MS. The sample size was 9 to 101 people. However, two studies that were performed online used 388 and 1568 samples. Prevalence of sexual dysfunction in all studies was reported from 31 to 92%. The type of studies performed was mostly cross-sectional, two were cohort studies and one was a case-control. In the case-control study, the index of sexual dysfunction prevalence was extracted in the case group. Erectile dysfunction (ED) is studied in 5 articles which mostly use the International Index of Erectile Function (IIEF-15) with 15 items. However, in some studies, scores less than 25 are considered ED and in others less than 21. In most of these studies, scoring was rated from 1 to 30, with a score of ≤ 25 as the dysfunction and 26 to 30 as normal. In one study, scoring ranged from 5 to 75 and score ≤ 45 was considered dysfunction and 46 to 75 as the normal range. One study used the 5-item International Index of Erectile Function-5 questionnaire (IIEF5), scoring ranged from 1 to 25, score ≤ 21 was considered dysfunction and 22 to 25 as the normal range. Three studies assessed sexual dysfunction and used the Multiple Sclerosis Intimacy and Sexuality Questionnaire-19 (MSISQ-19) with 19 items; five items dealt with primary, nine items secondary, and five items tertiary sexual dysfunction. Three studies assessed sexual dysfunction using the Szasz Sexual Functioning Scale in which score > 0 is considered dysfunction. The scale has five points: 0, sexually active as before and/or not experiencing sexual problems; 1, sexually less active than before, and/or now experiencing some sexual problems but not concerned; 2, sexually less active than before and/or now experiencing some sexual problems and concerned; 3, sexually inactive but still concerned; and 4, sexually inactive and not concerned (given up). Three studies assessed sexual dysfunction using the Multiple Sclerosis Quality of Life (MSQOL-54) sexual function scale. One study assessed sexual dysfunction using the Sexual Satisfaction Scale (SSS) that has 4 items in which scoring 4–24 and higher shows higher sexual dissatisfaction. Five studies used researcher-made different questionnaires and assessed sexual dysfunction or erectile dysfunction (ED) or sexual difficulties as listed in the table. The assessment method for the diagnosis of sexual dysfunction in 12 studies (60%) was self-administered questionnaires. The data obtained in interview methods (face-to-face structured interview, clinical interview, collected by the authors during consultation) are in 7 articles (35%), and just in one study (5%) physical examination were used beside postal questionnaires. In the current study, five studies just assessed ED in men with MS. They reported the prevalence of ED as 33.7 to 89% (Table 2).

**Table 2:** Main findings of 20 articles included in the current study

First author (reference number)	Year of publication	Country/city	Sample size	Age range and mean age	Duration of MS	Design of study	Outcome of study	Scale/index	The method of outcome assessment	Prevalence(***N*** of event)	Score of quality
Balsamo et al. [9]	2017	Italy	101	41.2 ± 11.6	11.5 ± 7.5	Cross-sectional	Erectile dysfunction (ED)	International Index of Erectile Function (IIEF-15) score ≤ 25	Self-administered questionnaire	In 75 patients (74.25%)	3
Barak et al. [10]	1996	Israel	9	35.4 ± 10.2	4.1 ± 2.7	Cross-sectional	Sexual dysfunction (SD)	Researcher-made questionnaire	Self-administered questionnaire	In 5 patients (55.5%)	4
Carnero Contentti et al. [11]	2019	Argentina	65	40.0 ± 8.0	7.09 ± 5.1	Cross-sectional	Erectile dysfunction (ED)	IIEF-15 scores ≤ 21	Self-administered questionnaire	In 49 patients (89%)	3
Çelik et al. [12]	2013	Turkey	45	37.4 ± 8.6	-	Cross-sectional	Sexual dysfunction (SD)	Multiple Sclerosis Intimacy and Sexuality Questionnaire-19 (MSISQ-19)	Self-administered questionnaire	In 22 patients (49%)	1
Darija et al. [13]	2015	Serbia/Belgrade	27	41.6 ± 6.9	9.2 ± 6.5	Cohort	Sexual dysfunction (SD)	Szasz sexual functioning scale (Szasz) score > than 0	Face-to-face structured interview	77.8% at baseline to 88.9% at the 6-year follow-up	2
Demirkiran et al. [14]	2006	Turkey	18	37.99 ± 8.9	7.3 ± 5.4	Cross-sectional	Sexual dysfunction (SD)	MSISQ-19	Structured face-to-face interview	In 14 patients (78%)	2
Finkelsztejn et al. [15]	2009	Brazil	17	43.5 ± 11.2	11.5 ± 6.7	Cross-sectional	Sexual dysfunction (SD)	Expanded Disability Status Scale (EDSS)–(??? not clear)	Data collected by the authors during consultation	31.1%	3
Ghezzi et al. [16]	1996	Italy	77	40.8 ± 9.0	9.1 ± 5.8	Cross-sectional	Erectile dysfunction (ED)	A subjective evaluation of their own sexual life	A clinical interview	In 26 patients (33.7%)	5
Hennessey et al. [17]	1999	South Glamorgan	68	50.2	-	Cross-sectional	Sexual dysfunction (SD)	Researcher-made questionnaire	Postal questionnaire and a physical examination	56 of 68(82%) reported deterioration in sexual activity	3
Lew-Starowicz and Rola [18]	2014	Poland	67	Range : 29–72; mean age: 49.9	15.5	Cross-sectional	Erectile dysfunction (ED)	IIEF-15 score ≤ 25	Self-administered questionnaire	ED was found in 52.9% of patients	3
Marck et al. [19]	2016	International	388	-	-	Cross-sectional	Sexual dysfunction (SD)	Multiple Sclerosis Quality Of Life (MSQOL-54) sexual function scale	Self-administered questionnaire—online survey	49.7 %	3
Nortvedt et al. [20]	2007	Hordaland County, Western Norway	14	Range: 17–53; mean: 32.9 ± 10	6.1	Cross-sectional	Dissatisfaction with their sexual functioning	Five of the 18 MS-specific questions constituting the sexual scale of the MSQoL-54	Self-administered questionnaire	50%	3
Orasanu et al. [21]	2013	A global registry	1568	38.47 ± 9.6	13.97 ± 9.3	Cross-sectional	Sexual activity and satisfaction (SAS)	MSISQ-19	Self-administered questionnaire—online survey	38.6% of males experienced at least 5 different types of severe symptoms	3
Redelman	2009	New South Wales, Australia	50	-	-	Cross-sectional	Sexual difficulties	Researcher-made questionnaire with 28 items	Self-administered questionnaire	74%	4
Tepavcevic et al. [22]	2008	Serbia/Belgrade	31	32.6 ± 8.5	9.0 ± 5.1	Cohort	Sexual dysfunction (SD)	Szasz Sexual Functioning Scale	Face-to-face structured interview	84% had one or more sexual disturbances	3
Tomé et al.´ [23]	2019	Brazil	41	Range: 22 to 66; mean: 40.7 ± 10.1	10.5 ± 7.3	Cross-sectional	Sexual dysfunction (SD)	IIEF-15 score ≤ 45	Interview	In 29 patients (74.4%)	3
Vazirinejad et al. [24]	2008	Derbyshire/England	38	51 ± 12.6	17 ± 10.8	Cross-sectional	Sexual problems	(MSQOL-54)	Self-administered questionnaire	In 29 patients (76%)	2
Winder et al. [25]	2018	Erlangen-Nuremberg	31	38.2 ± 11.2	4	Cross-sectional	Erectile dysfunction (ED)	5-item International Index of Erectile Function-5 questionnaire (IIEF5)—≤ 21	Self-administered questionnaire	In 14 patients (45.2%)	3
Zavoreo et al. [26]	2016	Croatia	42	35 ± 12	5.0 ± 1.5	Cross-sectional	Sexual dysfunction (SD)	Sexual Satisfaction scale (SSS)	Self-administered questionnaire	71%	3
Zorzon et al. [27]	1999	Italy/Trieste	38	40.8 ± 11.9	9.1 ± 6.4	Case-control	Sexual dysfunction (SD)	Szasz Sexual Functioning Scale	Face-to-face structured interview	92.1% had one or more sexual disturbances	3

**Fig. 3:**
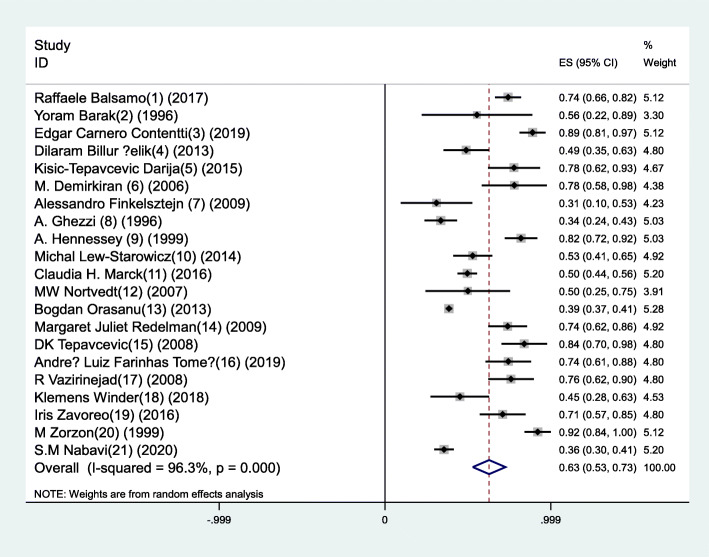
Prevalence of sexual dysfunction in men with MS and its 95% confidence interval (CI) in the considered studies based on the random effects model

The results of various studies showed that the pooled prevalence of sexual dysfunction in men with MS in all studies was 62.9% with 95% confidence interval 53 to 72.7% (Fig. 3).

## Discussion

Sexual dysfunction is one of the most frequent disorders in MS patients. This study tried to describe the prevalence of all types of sexual dysfunction in adult men with MS.

The prevalence of sexual dysfunction in adult men with MS was reported 31–92% in the articles included in the current study, and the pooled prevalence of sexual dysfunction in adult men with MS in all studies was 62.9%. High prevalence of sexual dysfunction in men with MS is consistent with Dupont’s study that reported a 47–75% sexual dysfunction prevalence in men with MS in a review article [28], and Schmidt et al. who reported a 64–91% sexual dysfunction prevalence rate in men with MS in a review article [29]. Seventy-four point four percent of men with MS had sexual dysfunction in Tomé et al.’s study [23]. Çelik et al. reported a 36.6 ± 13.3 sexual dysfunction prevalence rate in men with MS. [12]

In some of the studies, ED is reported as the most frequent dysfunction in men with MS. Contentti et al. reported ED as the most frequent symptoms with the prevalence of 23–91% in men with MS. [11] Balsamo et al. in their study reported the prevalence of ED as 74.2% which is high [9]. Tomé et al. reported ED in 66.7% of men with MS. [23] In the current study, five articles just assessed the prevalence of ED, reporting it to be 33.7 to 89% in range.

The present study found no association between age and prevalence of sexual dysfunction in men with MS. Some of the previous studies reported no significant relationship between erectile function deterioration and age [9, 25]. Çelik et al. reported, however, that MS patients with sexual dysfunction (men and women) were older than patients without sexual dysfunction. However, these differences were not statistically significant [12]. On the other hand, Hulter, Bakke, Valleroy, and Mattson reported significant relationship between sexual dysfunction and age [6]. In these studies, samples were men and women or just women. Experience of dyspareunia and diminished libido due to hormonal alterations in women that get older can affect sexual function, and it can explain this differences in the results [30].

As cited from previous studies [30], because of the progressive nature of MS, and the adverse effects of medication, longer diseases duration is considered a related factor of sexual dysfunction in MS patients [30]. However, in the current study, there were no significant relationship between duration of the disease and prevalence of sexual dysfunction. Winder et al. and Balsamo et al. reported no significant relationship between erectile function deterioration and duration of the disease in MS patients in their studies [25]. Çelik et al. reported that MS patients with sexual dysfunction (men and women) had longer disease duration than patients without sexual dysfunction. However, there was no statistical significance [12] and just a significant relationship was found between the secondary sexual dysfunction and diseases duration [12]. Our result about disease severity and prevalence of sexual dysfunction in adult men with MS showed that there is no significant relationship, like Winder’s study that reported no significant relationship between erectile function deterioration and the disease severity in MS patients [25]. Most studies that examined the prevalence of sexual dysfunction in MS patients are cross-sectional. In cross-sectional studies, it is not possible to control the intervention indicators. We are aware that some medications that are prescribed to treat MS could affect sexual function [9] and that depression is the most common psychiatric disorder in MS. [9] On the other hand, depression could also affect sexual function among healthy subjects [30]. Medications and depression are two examples of factors that can affect sexual function. In these circumstances, a research method that allows the control of intervention indicators like the randomized clinical trial, cohort, and case-control studies can provide more accurate information about sexual dysfunction in MS patients. In most of the articles reviewed in this study, the assessment method for the diagnosis of sexual dysfunction and obtaining data were self-reported with patients. This means that these data were not confirmed by direct evaluation of a specialist. Despite its important role in measuring the effects of various symptoms such as sexual dysfunction, the self-reported method for obtaining data is considered a limitation and is likely to have bias [11]. Moreover, patients’ responses to the questionnaires can be affected by their memory in recalling past information [31]. According to the results of the current study, this limitation exists in most studies that evaluate the sexual dysfunction prevalence in MS patients. The high prevalence of this assessment method can be due to the lack of clinical evaluation of sexual dysfunction in MS patients. Lack of clinical evaluation can be due to reasons including visit time limitation and patient discomforting [11]. Clinical evaluation of sexual dysfunction in MS patients can provide more accurate data.

To the best of our knowledge, this study is the first that describes the pooled prevalence of all types of sexual dysfunction in men with MS. However, there are some limitations in the current study. The first limitation was observed substantial heterogeneity across the studies included. Thus, meta-regression was used to determine potential sources of heterogeneity by included mean age, sample size, mean duration of MS, and the EDSS score. Second, the studies used different methods; the patients included in different studies aiming at reporting the prevalence of sexual dysfunction were not in the same MS duration. These items can induce observing bias in our results. Third, some of the included studies did not report the data according to men and women, separately, and we had to remove them from the current study.

## Conclusion

Sexual dysfunction is highly prevalent in adult men with MS. Due to the important role of sexual dysfunction in different (physical and mental) aspects of life, diagnosing sexual dysfunction in MS patients in clinics by specialists is suggested to be considered a necessity.

## Data Availability

All data generated or analyzed during this study are included in present published article.

## Abbreviations

MS: Multiple sclerosis
EDSS: Expanded Disability Status Scale
IIEF-15: International Index of Erectile Function
IIEF5: International Index of Erectile Function-5 questionnaire
MSISQ-19: Multiple Sclerosis Intimacy and Sexuality Questionnaire-19
MSQOL-54: Multiple Sclerosis Quality of Life
SSS: Sexual Satisfaction Scale
ED: Erectile dysfunction

## References

[1] Braback L, Kjellman NIM, Sandin A, Bjorksten B (2001). Atopy among schoolchildren in northern and southern Sweden in relation to pet ownership and early life events. Pediatr Allergy Immunol.

[2] MeSH Database. [Internet] https://www.ncbi.nlm.nih.gov/mesh/68020018. Accessed 17 June 2020.

[3] MeSH Database. [Internet] https://www.ncbi.nlm.nih.gov/mesh/68012735. Accessed 17 June 2020.

[4] Menkes DL, Castro-Borrero W (2014). Encyclopedia of the neurological sciences, second edn.

[5] Zhao S, Wang J, Liu Y, Luo Y, Zhu ZH, Li E (2018). Association between multiple sclerosis and risk of female sexual dysfunction: a systematic review and meta-analysis. J Sex Med.

[6] Redelman MJ (2009). Sexual difficulties for persons with multiple sclerosis in New South Wales, Australia. Int J Rehabil Res.

[7] Fakhri A, Morshedi H, Soleymanian A, Hosaini M (2014). Psychometric properties of Iranian version of male sexual function index. Jundishapur Sci Med J.

[8] Hoy D, Brooks P, Woolf A (2012). Assessing risk of bias in prevalence studies: modification of an existing tool and evidence of interrater agreement. J Clin Epidemiol.

[9] Balsamo R, Arcaniolo D, Stizzo M, Illiano E, Autorino R, Natale F (2017). Increased risk of erectile dysfunction in men with multiple sclerosis: an Italian cross-sectional study. Central Eur J Urol.

[10] Barak Y, Achiron A, Elizur A, Gabbay U, Noy S, Sarova-Pinhas I (1996). Sexual dysfunction in relapsing-remitting multiple sclerosis: magnetic resonance imaging, clinical, and psychological correlates. J Psych Neurosci.

[11] Carnero Contentti E, Pettinicchi JP, Caride A, López PA (2019). Sexual dysfunction in patients with multiple sclerosis from Argentina: what are the differences between women and men?. Sex Disabil.

[12] Çelik DB, Poyraz EÇ, Bingöl A, Idiman E, Özakbaş S, D. K (2013). Sexual dysfunction in multiple sclerosis: gender differences. J Neurol Sci.

[13] Darija KT, Tatjana P, Goran T, Nebojsa S, Irena D, Sarlota M (2015). Sexual dysfunction in multiple sclerosis: a 6-year follow-up study. J Neurol Sci.

[14] Demirkiran M, Sarica Y, Uguz S, Yerdelen D, Aslan K (2006). Multiple sclerosis patients with and without sexual dysfunction: are there any differences?. Mult Scler.

[15] Finkelsztejn A, Cristovam RDA, De Moraes GS, Lopes MGDSM, Da Silva AV, Garcia MS (2009). Clinical features of multiple sclerosis in the south of Brazil: a partial analysis. Arq Neuropsiquiatr.

[16] Ghezzi A, Zaffaroni M, Baldini S, Zibetti A (1996). Sexual dysfunction in male multiple sclerosis patients in relation to clinical findings. Eur J Neurol.

[17] Hennessey A, Robertson NP, Swingler R, Compston DAS (1999). Urinary, faecal and sexual dysfunction in patients with multiple sclerosis. J Neurol.

[18] Lew-Starowicz M, Rola R (2014). Sexual dysfunctions and sexual quality of life in men with multiple sclerosis. J Sex Med.

[19] Marck CH, Jelinek PL, Weiland TJ, Hocking JS, De Livera AM, Taylor KL, et al. Sexual function in multiple sclerosis and associations with demographic, disease and lifestyle characteristics: an international cross-sectional study. BMC Neurol. 2016;16(1):210. 10.1186/s12883-016-0735-8 PMC5097380 27814701

[20] Nortvedt MW, Riise T, Frugård J, Mohn J, Bakke A, Skår AB (2007). Prevalence of bladder, bowel and sexual problems among multiple sclerosis patients two to five years after diagnosis. Mult Scler.

[21] Orasanu B, Frasure H, Wyman A, Mahajan ST (2013). Sexual dysfunction in patients with multiple sclerosis. Mult Scler Relat Disord.

[22] Tepavcevic DK, Kostic J, Basuroski ID, Stojsavljevic N, Pekmezovic T, Drulovic J (2008). The impact of sexual dysfunction on the quality of life measured by MSQoL-54 in patients with multiple sclerosis. Mult Scler.

[23] Tomé ALF, Miranda EP, Júnior JDB, Bezerra CA, Pompeo ACL, Glina S, et al. Lower urinary tract symptoms and sexual dysfunction in men with multiple sclerosis. Clinics. 2019;74:1-7. 10.6061/clinics/2019/e713 PMC6399658 30892415

[24] Vazirinejad R, Lilley J, Ward C (2008). A health profile of adults with multiple sclerosis living in the community. Mult Scler.

[25] Winder K, Linker RA, Seifert F, Deutsch M, Engelhorn T, Dörfler A (2018). Insular multiple sclerosis lesions are associated with erectile dysfunction. J Neurol.

[26] Zavoreo I, Gržinčić T, Preksavec M, Madžar T, Bašić KV (2016). Sexual dysfunction and incidence of depression in multiple sclerosis patients. Acta Clin Croat.

[27] Zorzon M, Zivadinov R, Bosco A, Bragadin LM, Moretti R, Bonfigli L (1999). Sexual dysfunction in multiple sclerosis: a case-control study. I. Frequency and comparison of groups. Mult Scler.

[28] Dupont S (1995). Multiple sclerosis and sexual functioning - a review. Clin Rehabil.

[29] Schmidt EZ, Hofmann P, Niederwieser G, Kapfhammer HP, Bonelli RM (2005). Sexuality in multiple sclerosis. J Neural Transm.

[30] Azimi A, Hanaei S, Saharian MA, Mohamadifard M, Ramagopalan SV, Ghajarzadeh M (2019). Prevalence of sexual dysfunction in women with multiple sclerosis: a systematic review and meta-analysis. MAEDICA – J Clin Med.

[31] Nazari F, Shaygannejad V, Mohammadi Sichani M, Mansourian M, Hajhashemi V. Sexual dysfunction in women with multiple sclerosis: prevalence and impact on quality of life. BMC Urol. 2020;20(15):15. 10.1186/s12894-020-0581-2 PMC7035744 32085755

